# Effect of green tea on reward learning in healthy individuals: a randomized, double-blind, placebo-controlled pilot study

**DOI:** 10.1186/1475-2891-12-84

**Published:** 2013-06-18

**Authors:** Qiangye Zhang, Hongchao Yang, Jian Wang, Aiwu Li, Wentong Zhang, Xinhai Cui, Kelai Wang

**Affiliations:** 1 Department of Pediatric Surgery, Qilu Hospital, Shandong University, 107 Wenhuaxi Road, Jinan, Shandong 250012, China

**Keywords:** Green tea, Depression, Reward learning, Anhedonia

## Abstract

**Background:**

Both clinical and preclinical studies revealed that regular intake of green tea reduced the prevalence of depressive symptoms, as well as produced antidepressant-like effects in rodents. Evidence proposed that disturbed reward learning has been associated with the development of anhedonia, a core symptom of depression. However, the relationship between green tea and reward learning is poorly investigated. Our goal was to test whether chronic treatment with green tea in healthy subjects affects the process of reward learning and subsequently regulates the depressive symptoms.

**Methods:**

Seventy-four healthy subjects participated in a double-blind, randomized placebo-controlled study with oral administration of green tea or placebo for 5weeks. We used the monetary incentive delay task to evaluate the reward learning by measurement of the response to reward trial or no-reward trial. We compared the reaction time of reward responsiveness between green tea and placebo treatment. Furthermore, we selected Montgomery-Asberg depression rating scale (MADRS) and 17-item Hamilton Rating Scale for Depression (HRSD-17) to estimate the depressive symptoms in these two groups.

**Results:**

The results showed chronic treatment of green tea increased reward learning compared with placebo by decreasing the reaction time in monetary incentive delay task. Moreover, participants treated with green tea showed reduced scores measured in MADRS and HRSD-17 compared with participants treated with placebo.

**Conclusions:**

Our findings reveal that chronic green tea increased the reward learning and prevented the depressive symptoms. These results also raised the possibility that supplementary administration of green tea might reverse the development of depression through normalization of the reward function.

## Introduction

Green tea (*Camelia sinensis*) is one of the most popular beverages in both eastern and western world. Increasing evidence indicates that green tea extracts as well as their main component, the polyphenol epigallocatechin gallate (EGCG), has multiple health benefits, such as the anti-stress, anticancer and antioxidants effects [1-4]. In particularly, a recent investigation found that higher consumption of green tea led to a lower prevalence of depressive symptoms in elderly Japanese individuals [5]. Additionally, a preclinical study demonstrated that green tea exerted antidepressant-like effects in a mouse behavioral models of depression, and the mechanism may involve inhibition of the hypothalamic-pituitary-adrenal (HPA) axis [6]. These findings suggested that there might be a link between green tea consumption and the depressive symptoms. However, the underlying mechanism mediating in the treatment of green tea for depression in the clinic is poorly investigated.

Anhedonia is a core characteristic usually seen in depressed patients and is widely used to evaluate the treatment outcome of depression [7-9]. Impairment of reward learning is associated with a number of psychiatric disorders in humans including depression [10]. Anhedonia is also linked with reduced pleasure, altered motivation and disturbed reward learning [11,12]. Additionally, the reduced reward function was associated with the persistence of anhedonia in depressed subjects [13]. We hypothesize that chronic treatment with green tea would improve the reward learning compared with control subjects.

The monetary incentive delay (MID) task is usually used to assess the effort-related aspects of central reward processing for investigations of the relationship between depressive behavior and impairments in reward learning in depression [14]. Therefore, we hypothesized that green tea might play a critical role in modulation of reward learning and contribute to the phenotype of depressive symptom. The major goal of the current study was to evaluate the effects of green tea on reward learning and clinical outcome of depression after 5 weeks of treatment.

## Methods

### Participants

This study is a randomized, double-blind, placebo-controlled procedure. Seventy-four healthy subjects with both males and females were recruited by local advertisement aged between 18 and 34 years (mean age: 25.7±4.7years) in the study from March to November 2012. The study was approved by the Institutional Review Board of Shandong University (No. 1031). Before enrolment, participants underwent thorough screening, including a medical history interview, physical examination, typical tea consumption patterns, and clinical laboratory tests (including ECG and blood chemistry). The exclusion criteria were current or past history of psychotic disorders, a history of substance abuse or dependence, a history of neurological illness (sleeping disorder, epilepsy) or history of severe heart failure, abnormal ECG or other laboratory findings, and daily intake of tea more than three cups. Montgomery-Asberg depression rating scale (MADRS) and 17-item Hamilton Rating Scale for Depression (HRSD-17) were used to assess the depressive symptoms [15,16]. The scores of MADRS and HRSD-17 were measured before consumption of green tea and placebo as the baseline level and were measured again on the end of the experiment 5weeks later. Subjects in different treatment groups were similar in age, education as well as behavioral phenotype demonstrated by baseline MADRS total, HRSD-17 total scores (Table 1).Written informed consent was obtained before the experiment as approved by the Human Ethics Committee of Shandong University (No. 1031).

**Table 1:** Demographic characteristics of participants

	**Placebo**	**Green tea**
N	22	24
Gender (F/M)	12/10	11/13
Age (mean, sd)	24.55, 4.74 y	26.79, 4.48 y
Education (mean, sd)	16.14, 2.29 y	16.42, 2.67 y
MADRS (mean, sem.)	6.64, 0.35	6.29, 0.46
HRSD-17 (mean sem.)	7.32, 0.37	7.13, 0.39

*MADRS* Montgomery-Asberg Depression Rating Scale, *HRSD-17* 17-item Hamilton Rating Scale for Depression.

### Drug treatment

The green tea powder (Q/YFT0005S) was purchased from Yifutang Tea Co., Ltd. (Hangzhou, Zhejiang Province, China), and was packed at the weight of 400 mg per package. The participants were randomly divided into either green tea or placebo treatment group using random numbers when enrolled. The subjects were asked to take one package containing 400 mg of either green tea or cellulose three times each day for 5weeks. The placebo given to the control group comprised pure microcrystalline cellulose. It has been indicated from clinical pharmacokinetic and animal toxicological reports that consumption of green tea concentrated extracts on an empty stomach is more likely to lead to adverse effects than consumption in the fed state. Therefore, the package of green tea powder was dissolved in hot water and was taken 30min after each meal (three times per day) in the current investigation. The main chemical components of green tea are polyphenols (up to 20% and more of the dry weight) and caffeine (2-5%) [17]. This amount of caffeine consumption (20mg/day in one package of green tea powder) was not significantly associated with risk for health [18,19]. The major tea polyphenols and the amounts present in the powder used in the current study was shown in Table 2.

**Table 2:** Major tea polyphenols and the amounts present in the powder used in the current study

**Component**	**Amount**	**Method**
epigallocatechin-3-gallate (EGCG)	45.6%	HPLC
epigallocatechin (EGC)	16.7%	HPLC
epicatechin-3-gallate (ECG)	11.4%	HPLC
epicatechin (EC)	6.8%	HPLC
caffeine	0.6%	HPLC

*HPLC* High Performance Liquid Chromatography.

### Monetary incentive delay (MID) task

The MID task was applied with previous reports [14,20] with a minor modification and was consisted of 30 potentially reward trials, 30 no-reward trials, and 30 periods of fixation with an overall mean duration equal to trials. In total, trials lasted between 7.5 and 10.5 sec (mean: 9 sec). Thus, the total duration of the task was 13.5 min. Cues signaled potentially reward outcomes (red circle), or no-reward outcomes (green circle). Cue was presented with a variable duration for 4.5 – 9.5 sec (mean 7 sec) of each trial. On incentive trials, participants could win or avoid losing money by pressing the button during target presentation. When the target was pushed in a rewarding trial, participants earned 1 Yuan. Subjects were informed that they would receive one-third of the money they won during the MID in each session after completion of the study. Twelve practice trials were used to determine the shortest reaction time to a target of the subjects. The presentation time of the target was regulated according to the practice data to ascertain an equal feedback response and a similar total monetary reward for all subjects. The reaction time (ms) to reward and no-reward stimuli were recorded by the computer during each trial.

### Experimental procedure

The written informed consent was sent to the participants before the study. The scores of MADRS and HRSD-17 were measured as the baseline level. The next day, the participants were randomly divided into two groups based on their sequence of enrolment in the study and were treated with green tea powder or placebo, respectively. The green tea powder and the placebo (pure microcrystalline cellulose) were packed at the weight of 400 mg of each package. The green tea powder or placebo was dissolved in hot water and was taken 30 min after each meal (three times per day) for consecutive five weeks. Both the participants and the observers of the behavioral assessment were blind on the assignment of the experiment. One day after the last intake of green tea, participants were evaluated by MADRS and HRSD-17. Subsequently, the monetary incentive delay task was introduced into the participants and the reaction time (ms) to reward and no-reward stimuli were recorded by the computer.

### Data analysis

Data are expressed as mean ± SEM. The statistical analyses of the reward learning and depressive behavioral data in green tea and placebo-treated subjects were performed using repeated-measure ANOVA. Values of *p*<0.05 were considered statistically significant.

## Results

### Demographic characteristics

**Figure 1:**
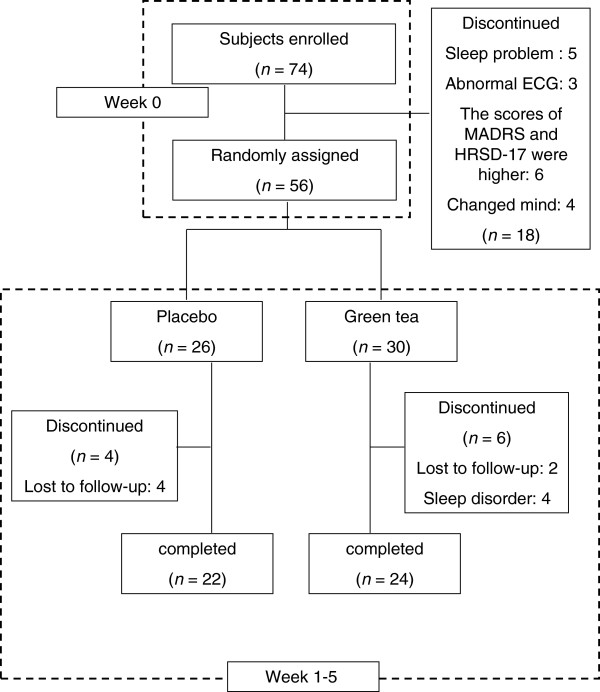
Procedure of subjects during the trial.

A total of 74 participants were enrolled in this study with similar gender, age, education and baseline MADRS total, HRSD-17 total scores (Table 1).There were 18 (24.3%) participants discontinued in the experiment for sleep problem (*n*=5), abnormal ECG (*n*=3), higher scores of MADRS and HRSD-17 (*n*=6), and mind changed (*n*=4). The rest 56 subjects were randomly assigned to different treatment receiving placebo (46.4%) or green tea (53.6%).There were 10 subjects discontinued for lost follow-up and sleep disorder. The 46 participants completed the study in placebo (*n*=22, 47.8%) and green tea (*n*=24, 52.2%) treatment (Figure 1).

### Effect of green tea on the reaction time in the monetary incentive delay task

**Figure 2:**
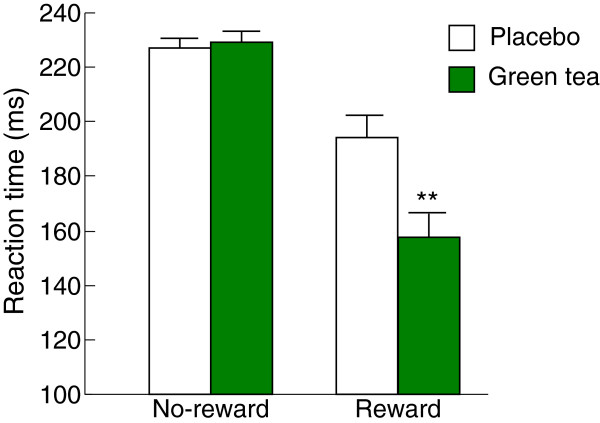
**Reaction time in the monetary incentive delay task after treatment with green tea (*n*** **= 24) or placebo (*n*** **= 22).** Data are expressed as mean ± SEM. Differences between placebo and green tea were assessed using Student’s *t* test. **p < 0.01, compared with placebo.

In the MID Task, ANOVA showed that green tea had significantly decreased reaction time in response to reward trial compared to placebo participants (*p*<0.01, Figure 2), suggesting that subjects treated with green tea showed a significantly increased reward learning. We also found that there were no differences for reaction time in the no-reward trials between placebo and green tea groups. It has been evidenced that reduced dopamine neurotransmission might contribute to the anhedonia and loss of behavioral incentive in depressive disorder [21,22], therefore it is important to examine the regulatory role of green tea on the brain circuitry activated by reward learning, such as the posterior and anterior cingulate, inferior parietal cortex, orbitofrontal, and superior frontal cortex [23,24]. The present data encourage further research to evaluate the neurobiological basis for potential regulation to reward response in affective illness.

### Effect of green tea on the scores of MADRS and HRSD-17

**Figure 3:**
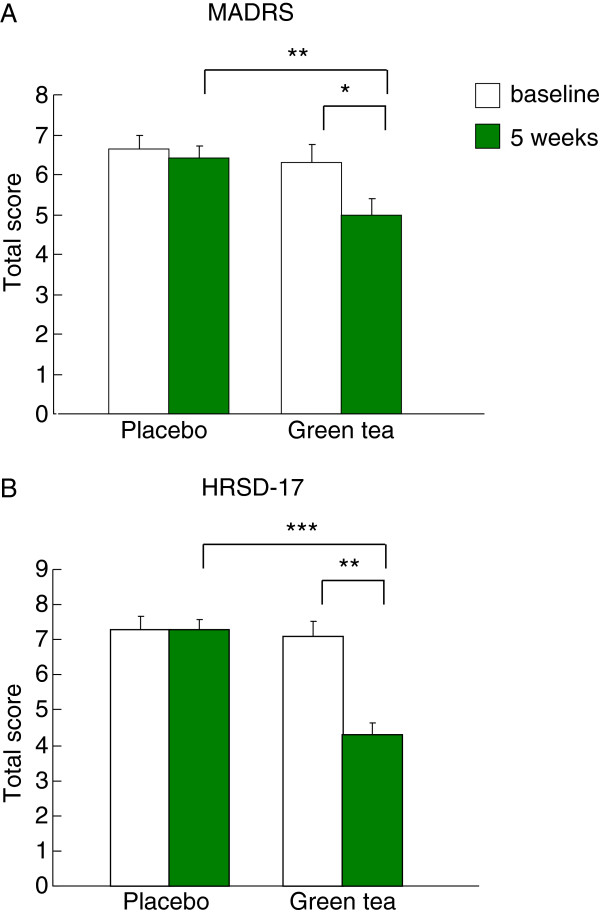
**Behavioral response to green tea and placebo in healthy subjects. (A)** MADRS and **(B)** HRSD-17 total scores were reduced by chronic treatment with green tea for 5 weeks. Data are expressed as mean ± SEM. Differences between green tea (*n* = 24) and placebo (*n* = 22) were assessed using Student’s *t* test. *p < 0.05, **p < 0.01, ***p < 0.001 compared with placebo. MADRS, Montgomery-Asberg Depression Rating Scale; HRSD-17, 17-item Hamilton Rating Scale for Depression.

To further identify the potential modulation of green tea on the reward function involved in the development of depressive symptoms, we conducted the MADRS and HRSD-17 to reflect the treatment outcomes. The data revealed that treatment with green tea for 5 weeks decreased both MADRS (5.00 ± 0.39 v.s. 6.29 ± 0.46, *p* < 0.05, Figure 3A) and HRSD-17 (4.33 ± 0.28 v.s. 7.13 ± 0.39, *p* < 0.001, Figure 3B) total scores compared with the baseline level. Whereas, there are no differences before and after 5-week treatment of placebo in both MADRS (6.41 ± 0.31 v.s. 6.64 ± 0.35, *p* > 0.05) and HRSD-17 (7.27 ± 0.29 v.s. 7.32 ± 0.37, *p* > 0.05) scores. In addition, the results showed that green tea produced significantly greater improvements on MADRS (5.00 ± 0.39 v.s. 6.41 ± 0.31, *p* < 0.01) and HRSD-17 (4.33 ± 0.28 v.s.7.27 ± 0.29, *p* < 0.001) total scores relative to placebo controls (Figure 3A, B). Administration of green tea for 5 weeks appears to be beneficial for the reward learning and the improved depressive symptoms. Additional long-term studies are warranted to confirm the relationship between reward learning ability and the recovery of the disorder in depressed patients.

## Discussion

Our results showed that oral administration of green tea increased the reward-learning ability compared with control group in healthy young volunteers. Moreover, participants treated with green tea showed reduced scores measured in MADRS and HRSD-17 compared with placebo. With the evidence that anhedonia influence reward decision-making, we propose that green tea would probably have the potential for normalization of anhedonia through improve reward learning and have implications for the prevention of depression.

A recent study showed that green tea extract treatment can reduce hypothalamic-pituitary-adrenal (HPA) axis hyperactivity in response to stress in mice [6]. Systemic administration of lipopolysaccharide (LPS) could induce depression in the forced swimming-induced despair behaviour model in mice. Pretreatment with green tea extract prevented LPS-induced immobility in a dose-dependent manner via COX-2 inhibition [25]. Clinically effective antidepressant medications exert their therapeutic actions partially by modulating HPA function through the regulation of receptor expression, subsequently ameliorating many of the behavioral disturbances associated with depressive-like states [26,27]. A further research is needed to determine the regulatory effect of green tea on glucocorticoids receptors expression and the target genes involved in the reward learning process and the improvement of depressive symptoms. Furthermore, a significant antidepressant-like effect was detected in mice that received a single intraperitoneal injection of green tea in the forced swim test when compared with the control [28]. A cross-sectional study revealed that higher green tea and coffee consumption was associated with a lower prevalence of depressive symptoms, suggesting that higher consumption of green tea, coffee and caffeine may confer protection against depression [29].

Anhedonia has long been presumed as a core feature of major depressive disorder based on the Feighner criteria in 1972 [30]. The American Psychological Association defines anhedonia as “report feeling less interest in hobbies, not caring anymore, or not feeling any enjoyment in activities that were previously considered pleasurable” [31]. Anhedonia and depressed mood are two required symptoms for a diagnosis of major depressive disorder [31,32]. It has been indicated previously that anhedonia is associated with impaired reward learning in depressed patients [9]. We confirmed that chronic treatment with green tea can improve the reward learning compared with baseline in healthy subjects at time of inclusion. Our results showed that oral administration of green tea increased the reward-learning ability compared with placebo.

It was proposed thirty years ago that dopamine deficiency and the reduction in dopamine (DA) transmission were involved in anhedonia, in individual’s experience of pleasure and in response to rewarding stimuli [33-36]. The mesolimbic and nigrostriatal DA system appears to be related primarily to reward system function and responsiveness to the environment [35]. In clinic, there is no effective intervention for anhedonia Although there are medications that alleviate several depressive symptoms, the current used pharmacotherapies (e.g., SSRIs) do not adequately address motivational and reward-processing deficits in depression [37-39]. It has been reported that the active component of green tea, EGCG, inhibited psychostimulants-induced hyperactivity in part by modulating dopaminergic transmission [40]. Additionally, the findings from epidemiological studies revealed that consumption of green tea was inversely correlated with neurodegenerative diseases including Parkinson's disease. The further investigation showed that EGCG produced a protective effect against dichlorodiphenyl-trichloroethane (DTT)-induced cell death, and that prior exposures to EGCG activate an endogenous protective mechanism in the dopaminergic cells, which is believed to play a causative role in the etiology of Parkinson's disease [41]. Moreover, EGCG also could reduce oxidative stress and neurotoxicity in different model systems of Alzheimer's disease and modulate the expression of cell survival and cell death genes [42].

In recent years, there has been considerable interest in investigating the neuroprotective effects of green tea polyphenolic constituent with its biological and pharmacological activities relevant to human health [43]. A previous study a mouse behavioral model showed that green tea exerts antidepressant-like effects, and the underlying mechanism may involve inhibition of the HPA axis [6]. Psychological stress activated HPA which resulted in the increasing release of glucocorticoids. It was also found that EGCG also could ameliorate the cognitive impairments induced by psychological stress possibly related with the declining glucocorticoids levels and the increasing contents of catecholamines and 5-HT [43]. Whether the increased reward-learning activity induced by chronic green tea administration is due to the regulation on neuroendocrinology systems, such as HPA function, requires future investigation.

Regular dietary intake of green tea can maintain a stable concentration in the body and may have valuable effects on health. However, drinking a beverage containing green tea extract too much can lead to hepatotoxicity [44-46]. The hepatotoxicity is probably due to EGCG or its metabolites which, under particular conditions related to the patient's metabolism, can induce oxidative stress in the liver [47-49]. Therefore, the toxicity should be taken into consideration in the condition of high oral consumption of green tea polyphenols. Additionally, the baseline amount of tea consumed in participants was screened before enrolment, and the participants whose daily intake of tea was more than three cups were excluded from the study. In the current study, the difference of baseline amount of tea in two groups was not significant. Therefore, the data in this experiment cannot be explained by an under measurement of effect.

In summary, our results indicate that chronic treatment of green tea increased reward learning compared with placebo in monetary incentive delay task and reduced total scores in Montgomery-Asberg depression rating scale and 17-item Hamilton Rating Scale for Depression. Reward-learning deficits contributed to the anhedonic symptoms and the treatment outcome of depression. The current findings suggest that reduced reward learning might associate with the presence of depression. The regulatory role of green tea on the reward learning raised the potential for development of new treatments for depression by selecting complementary options.

## Abbreviations

MID: Monetary incentive delay; MADRS: Montgomery-Asberg depression rating scale; HRSD-17: 17-item Hamilton rating scale for depression.

## Competing interests

The authors declare that they have no competing interests.

## Authors’ contributions

ZQY and LAW designed research, performed the statistical analysis and wrote the draft. YHC and WJ contributed to study design carried out the data analysis. ZWT, CXH and WKL participated in measurement of MADRS and HRSD-17 reporting and helped to draft the manuscript. All authors read and approved the final manuscript.
